# The antigenic evolution of influenza: drift or thrift?

**DOI:** 10.1098/rstb.2012.0200

**Published:** 2013-03-19

**Authors:** Paul S. Wikramaratna, Michi Sandeman, Mario Recker, Sunetra Gupta

**Affiliations:** Department of Zoology, University of Oxford, Oxford OX1 3PS, UK

**Keywords:** influenza, epitope, epidemiology

## Abstract

It is commonly assumed that antibody responses against the influenza virus are polarized in the following manner: strong antibody responses are directed at highly variable antigenic epitopes, which consequently undergo ‘antigenic drift’, while weak antibody responses develop against conserved epitopes. As the highly variable epitopes are in a constant state of flux, current antibody-based vaccine strategies are focused on the conserved epitopes in the expectation that they will provide some level of clinical protection after appropriate boosting. Here, we use a theoretical model to suggest the existence of epitopes of low variability, which elicit a high degree of both clinical and transmission-blocking immunity. We show that several epidemiological features of influenza and its serological and molecular profiles are consistent with this model of ‘antigenic thrift’, and that identifying the protective epitopes of low variability predicted by this model could offer a more viable alternative to regularly update the influenza vaccine than exploiting responses to weakly immunogenic conserved regions.

## 1. Introduction

Influenza A viruses are responsible for between three and five million cases of severe disease annually, and up to half a million deaths worldwide. These viruses are classified into subtypes on the basis of variation in their envelope glycoproteins, haemagglutinin (HA) and neuraminidase (NA), and the event of their replacement on a global scale is commonly referred to as an antigenic shift in the virus population. In the last 100 years, we have experienced three such shifts: in 1957, the H1N1 subtype that had been circulating since 1918 was replaced by H2N2; in 1968, H2N2 was replaced by H3N2; H1N1 was reintroduced in 1977 and has been cocirculating since with H3N2, although, in 2009, the current lineage was replaced by one derived from pre-existing swine, avian and human viruses. Each subtype, while in circulation, also undergoes a form of antigenic change culminating in the sequential dominance of antigenically distinguishable strains with very limited cross-sectional genetic diversity. The underlying process is generally visualized as a continuous and incremental transformation principally of the HA glycoprotein, and goes by the name of antigenic drift.

While it has considerable appeal as a verbal explanation for the epidemic behaviour of influenza, a formal link between the process of antigenic drift and patterns of influenza strain replacement is very difficult to make. This is because random mutation is much more likely to lead to a diffuse cloud of antigenic types on a variety of genetic backgrounds than the sequential emergence of discrete strains. A simple, but biologically unsatisfactory solution is to restrict the mode of mutation such that the virus population effectively travels in a pre-ordained straight line or circle [1,2]. The alternative explanation is that most mutants do not succeed, either because they are diffusing through genotypic space along phenotypically neutral networks [3] or because they are outcompeted by strains that have achieved greater antigenic distance from the preceding epidemic strains [4] or as a consequence of short-term strain-transcending immunological interference [5]. These additional assumptions can allow the virus population to progress in a linear manner through its available ‘antigenic space’ by counteracting the diffusive tendencies of antigenic drift. A common assumption here is that the potential for variation of the HA protein is extremely high and that long-term immune responses to HA are strain-specific, such that immunity to one antigenic type has no effect on any forms other than those that have very recently diverged from it. We have challenged this notion [6] by showing that epidemic behaviour of influenza can be readily explained by assuming that each strain elicits long-term partially cross-protective immune responses in addition to strain-specific immunity. This model—which we will henceforward refer to as the ‘antigenic thrift’ model (as suggested by Eddie Holmes)—departs from the conventional ‘antigenic drift’ hypothesis in a number of important ways: (i) there are a restricted number of unique but inter-connected antigenic states, (ii) the virus population has continuous access to these states, but (iii) most of these are unsuccessful owing to pre-existing partially cross-reactive immune responses in the host population.

We have no shortage of sequence data on influenza, but limited means as yet to use it to discriminate between competing hypotheses concerning the antigenic evolution of the virus population. Phylogenetic trees of influenza A exhibit a spindly structure that has commonly been misinterpreted as evidence of antigenic drift, but, in fact, simply indicates that the populations repeatedly pass through tight bottlenecks either as a result of selection or—as has recently been shown—as a straight-forward consequence of sampling sequences serially through time under neutral evolution [7]. It is clear that several of the models based on a process of antigenic drift [3–5] are capable of generating the observed trees; indeed, a principal focus of these efforts was to reproduce the ladder-like phylogeny of influenza A. It is as yet unknown, however, whether the antigenic thrift model is consistent with the phylogenetics of influenza. The antigenic relationships between different strains of influenza can also be determined using serological methods. An important focus of this paper is how the antigenic thrift model stands up to the scrutiny of sero-epidemiological analyses of the antigenic evolution of influenza.

We first provide a review of the antigenic thrift model; we then show how discriminating between epitopes of high and low variability provides a novel means of reconciling the dynamics of this model with empirical data on the antigenic evolution of influenza. Finally, we discuss how we may use improved serological techniques in conjunction with molecular methods to identify protective epitopes of low variability that may enable us to address the problem of influenza vaccination in a novel and practicable manner.

## 2. The antigenic thrift model

**Figure 1.**
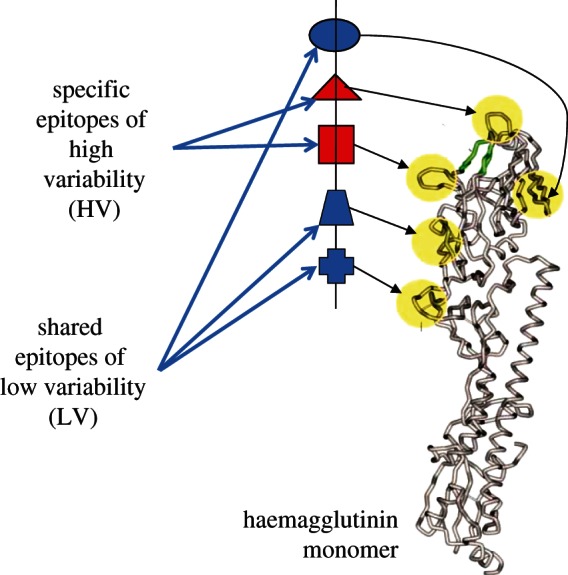
The antigenic thrift model is based on a multi-locus representation of the virus with each locus corresponding to an epitope region. This figure shows how these may locate to the known antigenic sites on a monomer of haemagglutinin (adapted from [71] & [72] with permission from OUP and NEJM, respectively).

Figure 1 provides a caricature of the fundamental assumptions of the antigenic thrift model in terms of how the various epitopes might map onto the structure of an HA monomer. In essence, the model combines highly variable, strain-specific epitopes (here visualized as surrounding the binding pocket) as well as epitopes of low to intermediate variability that are shared between strains. The model argues that immune responses against the latter are critical determinants of the protection against disease and onward transmission and drive the population dynamics of influenza in concert with antibodies directed at the uniquely strain-specific epitopes. Those individuals who have been exposed to a particular strain—as defined by a combination of these epitopes—have lifelong immunity to that same strain, but also have partial immunity (also lifelong) to strains related to it by virtue of possessing common epitopes. This network of cross-protection acts to limit the emergence of new antigenic types and can thus reconcile the high mutation rates of influenza with the dominance of a single antigenic type in each season.

**Figure 2.**
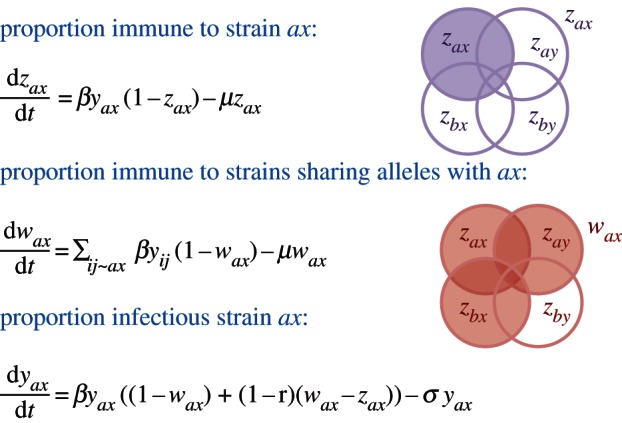
The structure of the antigenic thrift model is shown here with reference to a two locus and two allele system. A system of overlapping compartments can be used to indicate the proportions immune to each strain (*z*) and the proportion immune to antigenically related variants (*w*), from which the proportion infectious with this strain (*y*) can be deduced. In the diagram, *z_ax_* and *w_ax_* are indicated by purple and red shading, respectively. The notation *ij* ∼ *ax* indicates all strains sharing alleles with *ax*. The parameters **β** and 1/**σ**, respectively, define the transmission coefficient and infectious period of the virus, 1/**μ** corresponds to the life expectancy of the host population and **γ** measures the cross-immunity of a host gains from having seen a related but not identical variant.

Figure 2 summarizes the model structure using a simple schematic based on a system with only two relevant loci and alleles *a* and *b* at the first locus and *x* and *y* at the second. It is assumed that individuals who have been exposed to a particular strain (say *ax*, as shown in the diagram) are immune to further infection by the same strain, while those who have been exposed to strains that share alleles with it (in this example, *ay* and *bx*) have a reduced probability (1−**γ**) of transmitting the strain when infected; the parameter **γ** reflects the strength of allele-specific responses in preventing transmission. This structure can be easily generalized to multiple loci or epitopes with different levels of variability (i.e. number of possible alleles); we will henceforward use the notation {2,3,25}, for example, to indicate that there are three loci with 2, 3 and 25 alleles, respectively, and [i,j,k] to designate a particular strain or combination of alleles.

Mutation is not explicitly considered in this model. Instead, and since the model is deterministic, each possible antigenic variant is continuously present within the viral population. It is difficult to ascertain how this translates into an explicit mutation rate, but it does mean that at the precise moment a gap emerges in the network of host immunity, this gap can be exploited by any and all appropriate antigenic variants. Thus, it is this network of host immune responses, and not the mutational capability of the virus, that constrains observed antigenic diversity within the premise of antigenic thrift.

**Figure 3.**
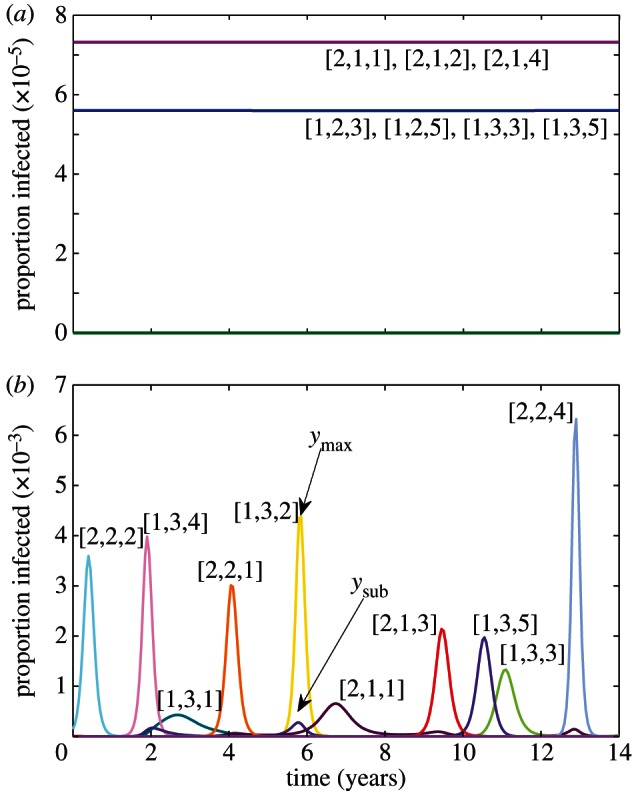
Strain dynamics within a {2,3,5} antigenic structure with (*a*) **γ** = 0.95 and (*b*) **γ** = 0.8 (**β** = 292; **σ** = 73; **μ** = 0.02).

**Figure 4.**
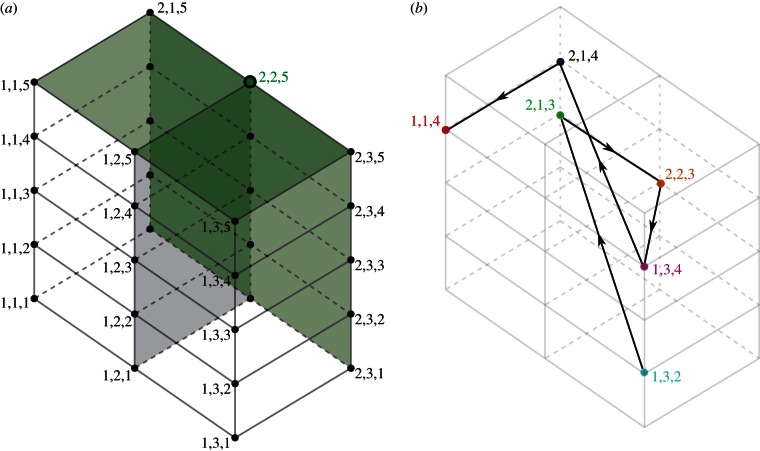
The {2,3,5} antigenic structure can be visualized in three-dimensional space with each axis representing a set of alleles at a particular locus, such that each point corresponds to a different antigenic variant. (*a*) Cross-reactivity against variant [2,2,5] extends within this space along the shaded green planes. (*b*) An example trajectory of the virus population through this space.

Multi-locus systems are capable of exhibiting two kinds of structuring, as shown in figure 3. At high levels of immune selection and provided that there is an equal number of possible alleles at each locus, discrete strain structure emerges with the stable maintenance of a set of strains that do not share alleles [8,9]. This discrete antigenic structure tends to break down in deterministic multi-locus models, when unequal numbers of allelic variants are instead possible at each locus but are recovered by the inclusion of stochastic processes [9]. At intermediate levels of immune selection, cyclical or chaotic strain dynamics (CSS) occurs [10]. We posit that the epidemic behaviour of influenza maps onto an area of CSS that exhibits high single strain dominance [6]. Figure 3*b* provides an example of this kind of dynamic for a {2,3,5} system; figure 4 traces a section of the antigenic trajectory of the virus population within a three-dimensional space that can be used to represent the relationships between all possible strains.

**Figure 5.**
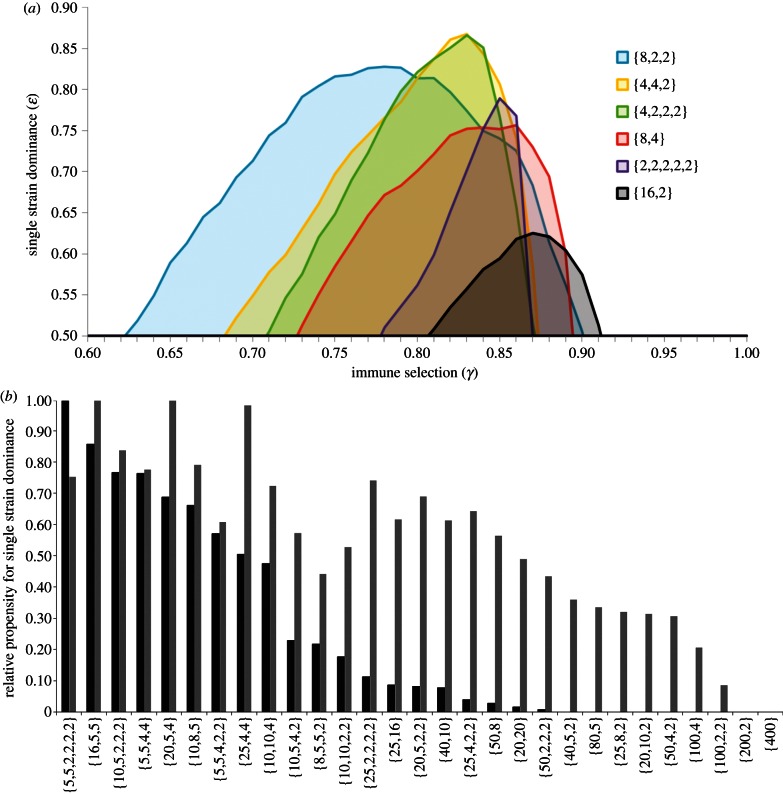
Impact of architecture of antigenic space on strong single strain dominance among different combinations of alleles and loci that produce a total of (*a*) 32 strains and (*b*) 400 strains. In (*b*), this is represented by the area under the curve, where **ɛ** > 0.5 (black bars) and **ɛ** > 0.1 (grey bars). Epidemics were identified by local maxima in total prevalence (**β** = 292; **σ** = 73; **μ** = 0.02).

Single strain dominance can be quantified by the measure of **ɛ** by comparing the relative prevalence of the two most common antigenic variants within single epidemics (figure 3), and then averaging across extended periods of time [6]. More formally, averaging across each of *P* epidemics:
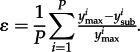


High **ɛ**, therefore, indicates strong single strain dominance as apparent for the antigenic evolution of influenza. Single strain dominance peaks at certain intermediate levels of **γ** [6], but also shows a dependence on epitope architecture. Figure 5*a* shows how different multi-locus systems, all with 32 total variants, differ in the region of **γ** where they exhibit strong single strain dominance. Interestingly, the combination that most favours strong single strain dominance here is {8,2,2}, while those that do least well are {16,2} and {2,2,2,2,2}, which respectively minimize and maximize connectivity. The complexity of the relationship between high **ɛ** and antigenic architecture is further demonstrated in figure 5*b* by comparing structures with 400 variants each. It is clear, nonetheless, that structures with high variability (HV) at every locus rarely tend to exhibit **ɛ** > 0.5 (even though they are in CSS) and a single-locus system with 400 alleles will not exhibit any oscillations whatsoever. Those combinations that perform well on this measure tend to contain some epitopes of low variability, but can also contain at least one highly variable locus.

## 3. Serological signatures of antigenic evolution

A common method for recording antibody levels in sera of either naturally or experimentally infected animals is the haemagglutination inhibition (HI) assay. HI assays exploit the ability of the influenza virus to bind to sialic acid receptors, and thus agglutinate avian and mammalian red blood cells [11]; the level of dilution at which a serum sample stops being able to prevent agglutination determines its HI titre. Published HI tables of human influenza A based on antisera raised in ferrets tend to show very high titres to the homologous isolate, but highly variable titres to temporally close isolates and, usually, a complete loss of reactivity against strains isolated more than a few years distant from the considered strain. This has commonly been interpreted as evidence of gradual antigenic drift, with each dilution step corresponding to an increase in antigenic distance. We have previously shown that such empirical data are consistent with the antigenic thrift model under the sampling scheme and multivariate analysis commonly used in their representation in two-dimensional antigenic space [6]. Indeed, the zig-zagging movement through antigenic space revealed by sophisticated cartographic methods applied to the evolution of H3N2 since 1968 [12] may be better explained by the antigenic thrift model. We have suggested that the apparent continuous increase in antigenic distance may be due to the censoring of entries between non-adjacent time-points, and that this signature of drift would disappear if accurate distance measures were available for all of the elements of the HI data matrix. This may be understood with reference to figure 4*b*: erasing the links between non-consecutive strains would have the effect of stretching out the trajectory, but nonetheless preserve some of the inherent transverse movement.

**Figure 6.**
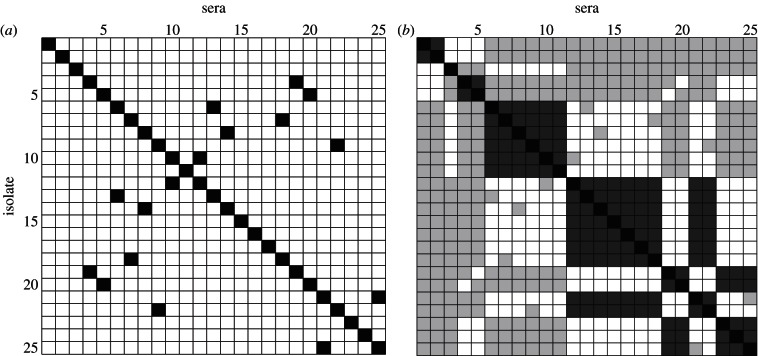
Pseudo HI tables constructed from the strength of cross-reactivity between the dominant variants of the *i*th and *j*th epidemics within a {2,3,25} antigenic space under different assumptions about the HI test: (*a*) HI only picks up the most variable epitope and strains only cross-react in the test (shown in black) if they are identical at this epitope and (*b*) HI titre depends on the number of shared epitopes with black/dark grey/light grey/white indicating epidemic strains that share 3/2/1/0 alleles, respectively. Epidemics were defined as local maxima in total prevalence more than 1.645 standard deviations from the mean, in line with [73]. (**γ** = 0.8, **β** = 292, **σ** = 73, **μ** = 0.02).

There are at least two alternative interpretations that reconcile the absence of reactivity by HI between non-adjacent time-points with the antigenic thrift model. The first is that HI assays selectively provide information on the unique strain-specific epitopes rather than the shared epitopes of limited diversity: in other words that the antibodies that prevent haemagglutination are directed only at the highly variable epitopes putatively clustering around the receptor-binding pocket as shown in figure 1. Figure 6*a* shows the antigenic relationships between strains within a {2,3,25} system as revealed by an assay that focuses solely on the third, highly variable, epitope region. A strong diagonal signature is observed, offering the impression of linear movement through antigenic space when in fact the population is actually zig-zagging within it in a manner analogous to that shown in figure 4*b*. Assuming instead that the magnitude of the HI titre depends on the precise number of shared epitopes (figure 6*b*), we can again find a strong diagonal signature, but with more evidence of clustering of similar antigenic variants in time, as seen in human HI data and by genetic analysis of influenza virus sequence evolution [13]. This emerges as a natural property of this model, owing to the time-scales at which epitope-specific immunity declines. In effect, as older hosts die, population-level immunity against the strains that they have specifically experienced wanes, thus creating gaps in the network of herd immunity that may be occupied by similar strains. This can lead to a sequence of antigenically related epidemics, until eventually a completely discordant allele combination is favoured and a cluster jump occurs. This is evident even in systems of low dimensionality such as the {2,3,5} example in figure 3*b*: the sequence [2,1,1] → [2,1,3] → [1,3,5] → [1,3,3] can be interpreted as a cluster transition between [2,1,*] → [1,3,*].

Another consideration in the interpretation of HI is that the laboratory animals used in generating the data may produce a response that is more focused towards the variable regions of HA than humans upon natural infection. HI assays are typically performed on post-infection antisera raised in ferrets and there is evidence suggesting that the cross-reactivities can be different to that of sera to the same isolate raised in mice and rabbits [14,15]. To our knowledge, no comparative study has been performed on post-infection sera taken from ferrets and humans or ducks and chickens, or indeed for many of the pairs of animals, where post-infection sera from the former are used to infer antigenic relationships in the latter.

Antigenic relationships between influenza strains can also be interrogated using microneutralization (MN) assays, where the virus is mixed with varying dilutions of serum and then inoculated into culture, and the presence of virus-specific antibodies in the serum is indicated by impaired or absent viral replication. HI titres correlate well with the results of MN assays [16], suggesting that both are detecting antibodies against highly variable epitopes putatively clustering around the receptor-binding pocket. However, discrepancies between the two assays—such as antisera with high neutralization titre but low or even absent HI titre or vice versa—have been reported for avian [17,18] and swine [19] influenza, and there are monoclonal antibodies that neutralize but do not inhibit haemagglutination and vice versa [20,21], suggesting that they do have different (although possibly overlapping) specificities. In theory, MN assays and other sophisticated serological techniques currently are in development [22], should be able to detect *additional* antibody responses against the epitopes of limited variability (LV) provided they are performed on the appropriate sera.

There is also growing evidence that antibodies targeting the stem region of HA (HA2) are capable of mediating protection from influenza infection. Such antibodies appear capable of recognizing a variety of different subtypes and strains, resulting also in protection of mice from lethal infection upon challenge (as reviewed by Yewdell [23]). In particular, monoclonal antibodies against HA2 have been shown capable of neutralizing drift variants of H3N2, and also protecting mice from challenge by the same variants [24]. Such responses do not appear to block viral attachment, and therefore do not exhibit cross-reactivity in the HI test, but may instead inhibit viral fusion [24,25]. The epitopes targeted by this class of antibody, may correspond to those of LV posited by the model of antigenic thrift, perhaps not least because escape from them can be precipitated by single point mutations in HA2 [26].

Epitopes of LV may also be located on NA; antibodies against these would act to prevent release of virus from the infected cell. The recent application of antigenic cartography methods to NA inhibition data reveals a high degree of asymmetry that is consistent with the antigenic thrift model. Sandbulte *et al.* [27] further suggest that antigenic evolution of NA may account for unexpected vaccine failures, where there is a good match based on HI data, highlighting the potential importance of more than just HI data in understanding the ways in which the virus can change to evade immune recognition.

## 4. Re-emergence of antigenic types

Perhaps, the most compelling evidence in support of the antigenic thrift hypothesis comes from the antigenic analysis performed in the wake of the 2009 H1N1 pandemic. Multiple serological studies have found evidence in the elderly of pre-existing antibodies that recognize the 2009 pandemic strain [28–35], and this appears to explain the low rates of disease in this age group during the pandemic [30,35–37]. Infection of both ferrets and mice with A/New Jersey/76 has been shown to confer strong cross-protection against challenge with 2009 pandemic H1N1, together with high cross-reactivity in the HI test [38–41]. In humans, receipt of the A/New Jersey/76 vaccine has been implicated in superior antibody responses against the 2009 strain [42]. H1N1 isolated prior to 1950 has also been shown to elicit significant, though less complete, protection upon challenge by pandemic H1N1 in both animal models, despite only weak cross-reactivity within HI and MN tests [38–41,43]. An inability of later seasonal isolates to protect against pandemic H1N1 challenge has been linked to glycosylation patterns on the globular head of HA, which may also explain why not all pre-1950 isolates appear to protect equally well [41].

Similar serological and clinical observations of cross-reactivity and clinical protection in the elderly have also been made in each of the previous three pandemics [44]. This could be explained by original antigenic sin (a phenomenon by which individuals continue to produce antibodies against the strain they were first infected with, even when challenged by a different strain/subtype), but this would still require a high degree of similarity between the returning strain and those to which they had been previously exposed.

There are hints of re-emergence of certain epitopes in antigenic analyses performed on H2N2 influenza, with a number of monoclonal antibodies raised against a 1957 strain cross-reacting strongly with a strain isolated in 1964, but not with a 1963 strain [45]. Antigenic analyses of influenza in pigs and birds are also suggestive of the re-emergence of antigenic types. Contractions in antigenic distance can be observed among populations of swine H1 viruses in the USA [46], and HI tables of H5N1 isolates from a variety of avian species demonstrate discordances in cross-reactivity [47] that are more easily explained by recycling of variants than incremental accrual of antigenic distance.

## 5. Discussion

Many features of the epidemiology of influenza can be explained by assuming that neutralizing antibodies act upon shared epitopes of LV as well as upon epitopes of HV that may be unique to a particular epidemic strain. We argue here that the use of HI assays has focused our attention on HV epitopes, but immune responses against these alone cannot produce a sequential emergence of antigenic types. We propose that the structuring of the virus population is achieved principally through immune responses against the LV epitopes which are not adequately represented in HI tables as they have a limited role in the binding of the virus to RBC. The existence of these additional LV epitopes is consistent with the observation that clinical protection and HI titre are logarithmically related, with limited improvement in protection beyond a certain (fairly low) titre [48], and also explains why high vaccine efficacy is sometimes observed even when HI data indicate that the incoming influenza strain has changed [49]. The results of early studies showing induction of superior HI titres with inactivated vaccines, but inferior protection from disease and shedding when compared with live vaccines [50] can be justified within this framework, if the latter induce a broader response that includes the LV epitopes. It is also tempting to speculate that some of the effects of original antigenic sin may be attributed to the skewing of antibody responses towards LV epitopes in later infections. In other words, a naive individual would produce antibodies to both HV and LV epitopes, but selectively towards LV epitopes in subsequent infections since they are shared between strains. They would thus maintain a strong HI response to the original strain, but may not show high HI titres to more recent infections. A recent Japanese study [51] showing that sera from young children were prone to recognize only the antigenic site B1 of the HA1 region of H3, in contrast to older individuals who had broad recognition, supports this idea.

Our model is not unique in invoking multiple components of immunity, but differs critically from other frameworks in that the targets of immunity divide between HV and LV epitopes, which both elicit strong specific lifelong immunity and do not include epitopes that induce short-term non-specific immunity (as in [5]). The validity of the antigenic thrift model rests on the existence of LV epitopes: identifying these would thus allow us to discriminate between competing hypotheses concerning the antigenic evolution of influenza (although they are by no means mutually exclusive), but, more importantly, could form the basis of a new vaccine that would release us from our dependence on monitoring change in the HV epitopes, and could complement the use of both antibody and T-cell-based vaccines towards fully conserved but weakly immunogenic epitopes that are currently under development [52].

How do we go about looking for LV epitopes, if they are not visible by HI? We anticipate that the dissection of antibody responses in human sera using MN assays, in conjunction with other more advanced techniques in the pipeline [22], will assist in identification, but we can also use molecular sequencing methods? It has long been recognized that the virus possesses a number of discrete antigenic sites [53], and several studies indicate that the number of amino acid differences in these sites is a better predictor of vaccine efficacy than HI data from ferrets [54,55]. We have previously highlighted that there is LV at 18 amino acid positions that have been identified by Bush *et al.* [56] as being under positive selection in H3N2; several of these are represented among the key immunodominant positions identified by pairwise comparison of consecutive epidemic strains [12,57] and by analysis of change in net charge [58]. Amino acid changes in the HA epitopes of H2N2 [45], H1N1 [59] and highly pathogenic H5N1 [60] also seem to be subject to strong restrictions. Epistatic interactions between sites, as documented by Kryazhimskiy *et al.* [61], may act to further reduce the potential nodes within antigenic space that may be occupied by the virus or favour a particular combination even under a very slight increase in transmissibility [62]. Sequence similarities between epitopes of 1918 H1N1 influenza and the 2009 pandemic strain have been used to justify pre-existing immunity to the latter [63,64]; similar arguments have also been made for the presence of neutralizing antibodies among individuals born before 1957 by comparing the HA sequences of 1957 and 2009 H1N1 strains [65]. Many of these studies rely on HI assays to discriminate between strains: the antigenic thrift model would predict that this method would selectively emphasize the role of mutations near the receptor-binding sites and, indeed, this seems to be the case in a recent study [63]. Rudneva *et al.* [63] also recorded some discordance between HI and ELISA studies, suggesting that the use of HI may not be sufficient to pick-up the LV epitopes. Newer methods such as panning of whole-genome-fragment phage display libraries (GFPDL) with convalescent human sera [66], tend by contrast to direct our attention towards weakly immunogenic conserved epitopes, but may be deployed to pick out LV epitopes with finer resolution of HA and NA gene fragments. It is also important that these analyses include considerations of effects of glycosylation, alteration of biophysical properties [67], complex interactions between residues at antigenic sites both within and between HA and NA [61,68,69] and potential effects of antibody interference [70]. Combining these techniques to elucidate the functional repertoire of human antibodies to influenza will be invaluable in resolving to what extent its epidemiology is determined by epitopes of LV, and whether these may be used to confer broader protection.

## Note added in proof

Zinder *et al*. [74] have recently shown that the phylodynamics of influenza can be readily generated within a similar framework with a number of epitope regions of limited diversity, under a somewhat different cross-immunity structure, provided it is also mutation-limited. This suggests that it will be difficult to discriminate between competing hypotheses of antigenic evolution on the basis of their ability to generate realistic phylodynamic patterns.
